# Predictive Value of Combined Positive Score and Tumor Proportion Score for Immunotherapy Response in Advanced NSCLC

**DOI:** 10.1016/j.jtocrr.2023.100532

**Published:** 2023-05-25

**Authors:** Ezgi B. Ulas, Sayed M.S. Hashemi, Ilias Houda, Adem Kaynak, Joris D. Veltman, Marieke F. Fransen, Teodora Radonic, Idris Bahce

**Affiliations:** a Department of Pulmonary Medicine, Amsterdam University Medical Centers, Location VU University Medical Center, Cancer Center Amsterdam, Amsterdam, The Netherlands; b Department of Pathology, Amsterdam University Medical Centers, Location VU University Medical Center, Cancer Center Amsterdam, Amsterdam, The Netherlands

**Keywords:** Non–small cell lung cancer, Immunotherapy, Predictive biomarker, PD-L1, Combined positive score

## Abstract

**Introduction:**

In advanced-stage NSCLC, tumor proportion score (TPS) is typically used to predict the efficacy of immune checkpoint inhibitors (ICIs). Nevertheless, in other cancer types, the combined positive score (CPS), which covers programmed death-ligand 1 (PD-L1) expression on both tumor and surrounding immune cells, is used. We investigated the predictive value of CPS in comparison to TPS in advanced NSCLC.

**Methods:**

A monocenter, retrospective study was performed in patients with advanced NSCLC treated with ICI monotherapy between 2015 and 2021. Hematoxylin and eosin and PD-L1 were stained on baseline tumor biopsy samples to score PD-L1 by both TPS and CPS. Positivity for TPS and CPS was defined as a score of 1% or above. Progression-free survival and overall survival (OS) were assessed for TPS and CPS scores.

**Results:**

Among the 187 included patients, PD-L1 positivity was found in 112 patients (59.9%) by TPS and 135 patients (72.2%) by CPS. There was no significant difference in OS between TPS^−^ and TPS^+^ patients (*p* = 0.20). Nevertheless, CPS^+^ patients did have a longer OS than CPS^−^ patients (*p* = 0.006). OS was superior in both TPS^−^/CPS^+^ and TPS^+^/CPS^+^ as compared with TPS^−^/CPS^−^ cases (*p* = 0.018 and *p* = 0.015, respectively), whereas no considerable differences in OS were found between TPS^−^/CPS^+^ and TPS^+^/CPS^+^ cases.

**Conclusions:**

This retrospective real-world population study revealed that CPS differentiated OS better than TPS in patients with advanced NSCLC with ICI monotherapy. Remarkably, this was driven by the performance of the TPS^−^/CPS^+^ subgroup, indicating that CPS may be a better predictive biomarker for ICI efficacy.

## Introduction

The approval of immune checkpoint inhibitors (ICIs) targeting programmed cell death protein 1 and programmed death-ligand 1 (PD-L1) has dramatically changed the treatment landscape for patients with NSCLC. Numerous studies in the metastatic setting have found higher response rates and more favorable survival outcomes when patients were treated with ICI monotherapy or ICI in combination with chemotherapy as compared with the conventional chemotherapy alone.^1^, ^2^, ^3^, ^4^ Despite these promising improvements, response to ICI treatment is not guaranteed in all patients,^5^ highlighting the need for predictive biomarkers.

One such biomarker that is currently used in routine clinical practice in patients with NSCLC is the expression of PD-L1 on tumor cells, that is, the tumor proportion score (TPS). Tumors with TPS scores of 50% or above seem to have a higher likelihood to respond to ICI treatment alone, whereas those with a lower TPS percentage could best be treated with a combination of an ICI and chemotherapy.^6^ In contrast to NSCLC, other cancer types are scoring PD-L1 expression through the combined positive score (CPS), which covers the PD-L1 expression on both the tumor cells and the immune cells in the tumor microenvironment. For example, in both gastric carcinoma and head and neck squamous cell carcinoma, the prediction of tumor response to ICI treatment was found to improve when immune cells were included in the PD-L1 scoring, as revealed in the KEYNOTE-012 and KEYNOTE-055 studies, respectively.^7^^,^^8^ Therefore, CPS rather than TPS is being used in these cancer types.

Whether CPS could be a better predictive biomarker than TPS in NSCLC is unclear, as the available data in the literature are very limited. Therefore, this retrospective real-world population study aimed to evaluate both TPS and CPS scores to correlate them with survival outcomes in patients with metastatic NSCLC treated with ICI monotherapy.

## Materials and Methods

### Study Population

Patients with advanced-stage NSCLC who received ICI treatment between 2015 and 2021 at the Amsterdam University Medical Centers were screened to identify eligible patients for this monocenter retrospective study. Patients who received at least one cycle of monotherapy ICI treatment and had a pretreatment biopsy available for immunohistochemistry (IHC) staining were considered eligible for this study. All clinical data were collected from the patient’s medical records.

Ethical approval was obtained by the institutional review board, and all patients consented to the collection of both their clinical and pathologic data (Institutional Review Board/METc VUmc numbers 2017.333 and 2017.545).

### Clinical Data

The collected clinical data included patient characteristics such as sex, age, performance status (according to the Eastern Cooperative Oncology Group), and smoking status. In addition, data on pathologic features such as histologic type and origin sites of baseline biopsies were collected. For survival analysis, data on the overall survival (OS) and progression-free survival (PFS) were used. OS was defined as the time interval between start of ICI treatment and death. PFS was defined as the time from start of ICI treatment to clinical or radiologic disease progression, or death if occurring first. Radiologic disease progression was determined by using Response Evaluation Criteria in Solid Tumors version 1.1. Patients who were progression free or alive at the last follow-up (October 1, 2022) were censored.

### Immunohistochemistry

For each case, routine hematoxylin and eosin and PD-L1 staining was performed by IHC on formalin-fixed, paraffin-embedded samples with representative tumor tissue consisting at least 100 tumor cells. PD-L1 staining was performed using the laboratory-developed test with PD-L1 clone 22C3 stained on the Dako Autolink stainer. The test was validated against the pharmDx kit and performed on the same machine, as described before.^9^, ^10^, ^11^ Baseline bone biopsy samples were decalcified using the ethylenediamine tetra-acetic acid decalcification method, as described before.^12^ A validation series of PD-L1 staining was performed to confirm that ethylenediamine tetra-acetic acid–based decalcification had no influence on PD-L1 IHC. Further elaboration on the PD-L1 staining is described in the Supplementary Methods.

### Assessment of PD-L1 Expression

Expression of PD-L1 by TPS and CPS was assessed by TR and EBU together in a test cohort (n = 50), afterward separately in the rest of the cohort, and the discrepant cases were discussed together over the microscope for the consensus score.

TPS and CPS were scored according to previously reported methods.^7^^,^^8^^,^^13^ TPS was defined as the proportion of viable tumor cells that reveal PD-L1 staining of any intensity. Tumor cells were considered positive in case of membranous staining, of which both partial and complete staining were accepted in several patterns such as circumferential and basolateral. If only cytoplasmic staining of tumor cells was observed, these cells were considered to be negative to PD-L1 expression.

CPS scoring of PD-L1 IHC was performed as widely reported elsewhere.^14^ Only inflammatory cells that were present in the same tissue fragment as tumor cells were counted. CPS was calculated by means of the following formula: (PD-L1–positive tumor cells + PD-L1–positive mononuclear inflammatory cells) / (total tumor cells) × 100. According to the used calculations, CPS scoring outcomes could exceed 100, yet the maximum possible score was fixed at 100.

### TPS and CPS Cutoff Thresholds

In clinical practice, the TPS cutoff values of 1% and 50% are used for PD-L1 scoring in NSCLC and the determination of treatment strategy, on the basis of the results of several randomized controlled trials.^15^^,^^16^ For other cancer types in which CPS is the official standard, the thresholds of less than 1%, 1% to 9%, 10% to 19%, and more than or equal to 20% are often used.^17^ Because of the absence of CPS-specific thresholds in NSCLC, these cutoff values were adapted in the present study along with the known thresholds of less than 1%, 1% to 49%, and more than or equal to 50%. TPS and CPS positivity was defined as 1% or more.

### Statistical Analyses

Frequencies and percentages were reported for categorical variables and median and range for continuous variables. Survival curves for OS and PFS were plotted using the Kaplan-Meier method. Differences in survival probabilities were assessed by using the log-rank test, and the Cox regression model was used to estimate the hazard ratios (HRs). Cohen’s kappa coefficients were used for interobserver agreement analysis. To assess differences in baseline characteristics, univariate analyses of variance and chi-square (χ^2^) tests were performed. All statistical analyses were performed using IBM SPSS Statistics version 26 (SPSS, Chicago, IL). All *p* values were two sided, and *p* value less than 0.05 was considered statistically significant.

## Results

### Patient Enrolment and Characteristics

**Figure 1:**
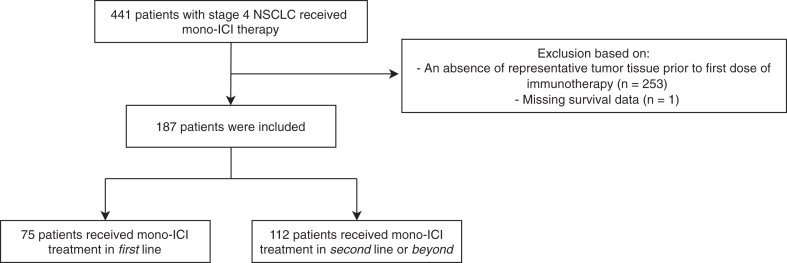
Flowchart of patient inclusion. ICI, immune checkpoint inhibitor.

In this real-world retrospective study, data were collected from 441 patients with NSCLC who received ICI treatment at one institution, of which 254 were excluded due to (1) an absence of representative tumor tissue before first dose of immunotherapy or (2) missing survival data. Ultimately, 187 patients were included in the study. The flowchart depicting the patient inclusion is found in Figure 1.

The included group of patients consisted of 95 males (50.8%), and the median age was 64 (range: 31–89) years. There were 123 patients who had an adenocarcinoma (65.8%), and most included patients received ICI therapy in the second line or beyond (69.9%). The clinicopathologic characteristics at baseline are presented in Table 1. An additional table on clinicopathologic characteristics, including oncogenic alterations, is found in Supplementary Table 1.

**Table 1:** Baseline Clinicopathologic Characteristics of the Included Patients (N = 187)

Characteristic	n (%)
Median age = 64 (32–89) y	
Sex	
Male	95 (50.8)
Female	92 (49.2)
ECOG PS	
0	39 (20.9)
1	105 (56.1)
2	31 (16.6)
3	10 (5.3)
4–5	0 (0)
Unknown	2 (1.1)
Smoking status	
Active	61 (32.6)
Former	109 (58.3)
Never	11 (5.9)
Unknown	6 (3.2)
Histologic type	
Adenocarcinoma	123 (65.8)
Squamous cell carcinoma	42 (22.5)
NSCLC NOS	12 (6.4)
LCNEC	7 (3.7)
Sarcomatoid carcinoma	2 (1.1)
Adenosquamous carcinoma	1 (0.5)
Immune checkpoint inhibitor	
Nivolumab	125 (66.8)
Pembrolizumab	60 (32.1)
Atezolizumab	2 (1.1)
Line of treatment	
First	75 (40.1)
Second or beyond	112 (59.9)
Site of tissue origin used for PD-L1 staining	
Lung (primary)	94 (50.3)
Lymph node	23 (12.3)
Liver	21 (11.2)
Soft tissue	14 (7.5)
Bone	10 (5.3)
Adrenal	7 (3.7)
Pleura	5 (2.7)
Cerebrum	4 (2.1)
Lung (metastasis)	2 (1.1)
Skin	2 (1.1)
Other	5 (2.7)

ECOG PS, Eastern Cooperative Oncology Group performance score; LCNEC, large cell neuroendocrine carcinoma; NOS, not otherwise specified; PD-L1, programmed death-ligand 1.

### PD-L1 Expression Measured by TPS and CPS

**Figure 2:**
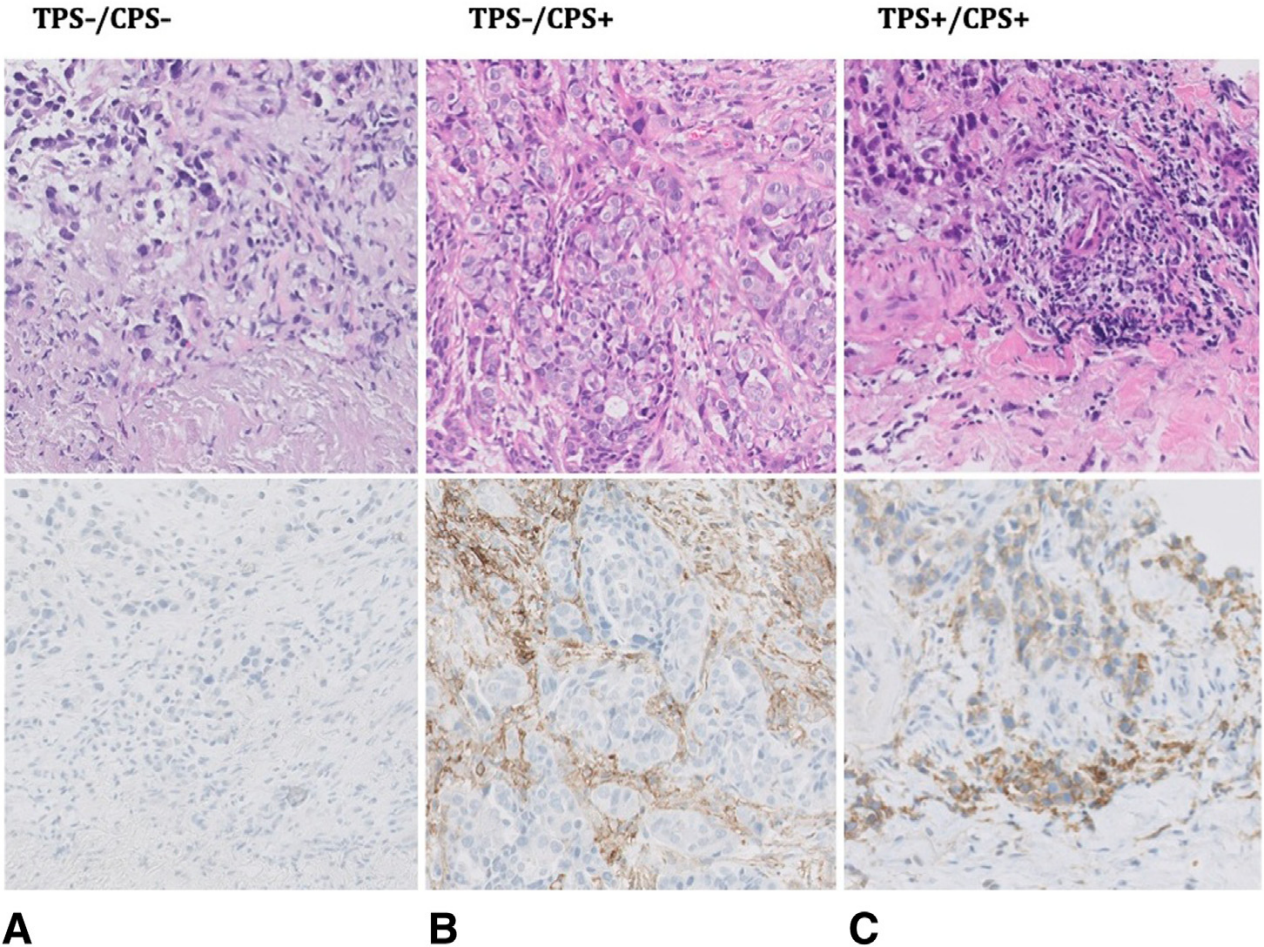
(*A*) Bar charts revealing the distribution of the TPS/CPS scoring outcomes. (*B*) Scatterplot revealing the distribution of the TPS/CPS scoring outcomes. CPS, combined positive score; TPS, tumor proportion score.

**Figure 3:**
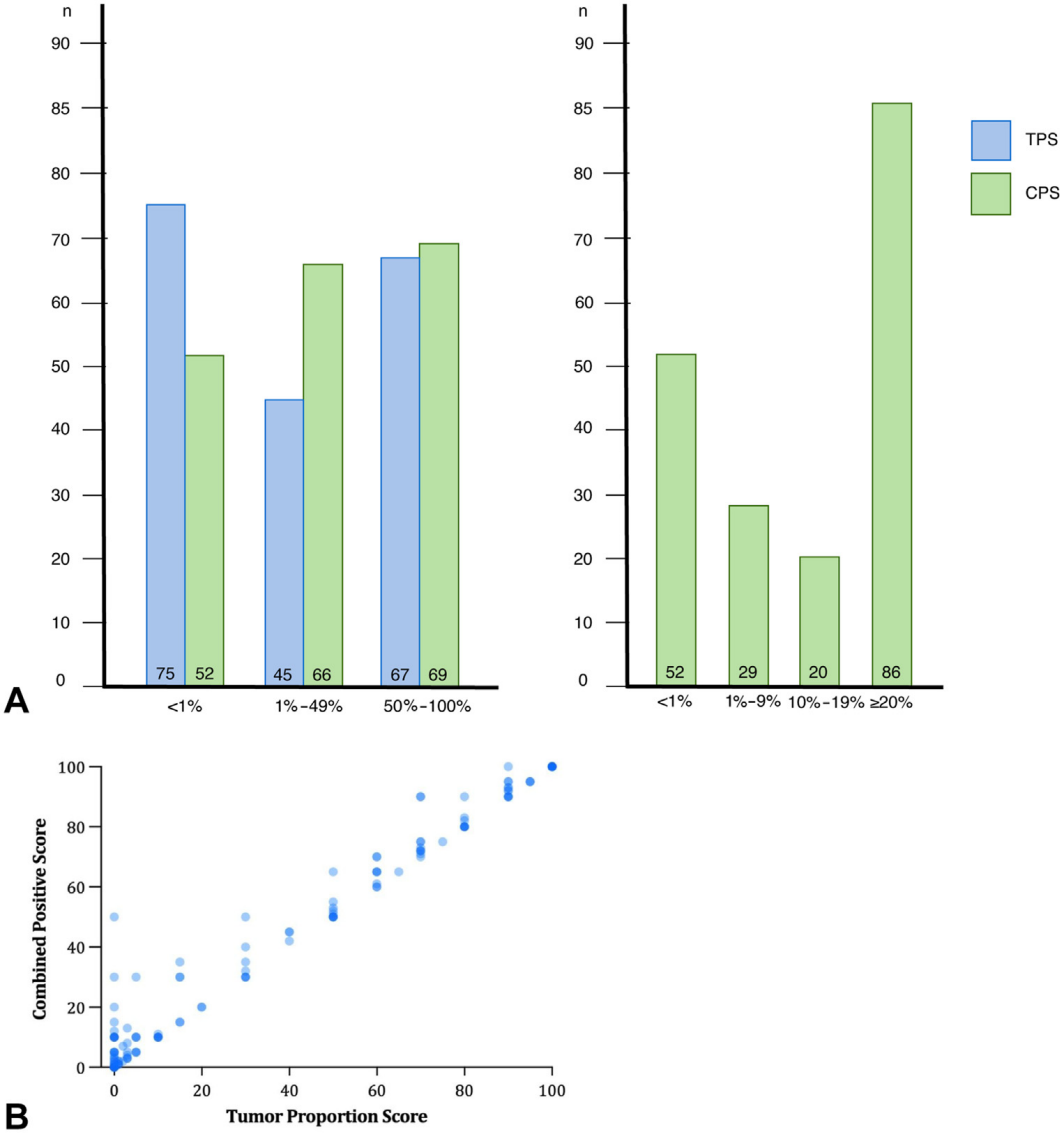
Examples of PD-L1 evaluation by TPS and CPS. (*A*) Case with both TPS and CPS-negative (TPS 0%, CPS 0%). (*B*) Case with TPS-negative and CPS-positive (TPS 0%, CPS 50%). (*C*) Case with both TPS and CPS-positive (TPS 30%, CPS 50%). CPS, combined positive score; TPS, tumor proportion score.

Any TPS positivity (≥1%) was found in a total of 112 patients (59.9%) (<1%, 75; 1%–49%, 45; ≥50%, 67) and any CPS positivity (≥1%) in 135 patients (72.2%) (<1%, 52; 1%–9%, 29; 10%–19%, 20; ≥20%, 86). Figure 2*A* and *B* reveal the distribution of the 187 histologic samples assessed for both TPS and CPS, respectively. Interestingly, we found CPS positivity in 23 patients despite a negative TPS score (1%–9%, 12; 10%–19%, 8; ≥20%, 3). Next to the survival analyses for the predefined thresholds of TPS and CPS, patients were divided into the following three clinically relevant groups: (1) the negative TPS and negative CPS subgroup, (2) the negative TPS and positive CPS subgroup, and (3) the TPS-positive and CPS-positive subgroup. Figure 3 illustrates examples of these three PD-L1 expression subgroups. The interobserver agreement analysis, as found in Supplementary Table 2, resulted in Cohen’s kappa coefficients of 0.79 and 0.71, which indicates substantial agreement between the two observers.

#### Progression-Free Survival

Supplementary Figures 1A-D and 2A and B illustrate the correlation of different tumor PD-L1 expression scores with PFS. The median PFS in the TPS-negative group was 2.8 months versus 4.1 months in the TPS-positive group (HR = 0.86, 95% confidence interval [CI]: 0.63–1.19, *p* = 0.37). The median PFS was 1.9 months in the CPS-negative group versus 4.1 months in the CPS-positive group (HR = 0.72, 95% CI: 0.51–1.02, *p* = 0.065).

The median PFS was 2.8, 2.1, and 6.0 months in the TPS less than 1%, TPS 1% to 49%, and TPS more than or equal to 50% groups, respectively. When divided into CPS less than 1%, CPS 1% to 49%, CPS more than or equal to 50%, the median PFS was 2.1, 2.7, and 6.0 months, respectively. The median PFS was 1.9, 3.6, and 4.1 months in the TPS^−^/CPS^−^, TPS^−^/CPS^+^, and TPS^+^/CPS^+^ groups, respectively. Using the four prespecified thresholds of CPS (<1%, 1%–9%, 10%–19%, ≥20%), the median PFS was 1.9, 1.6, 4.6, and 5.3 months, respectively. Separate PFS analyses performed for first versus second line or beyond are found in Supplementary Figure 3*A* and *C*.

#### Overall Survival

**Figure 4:**
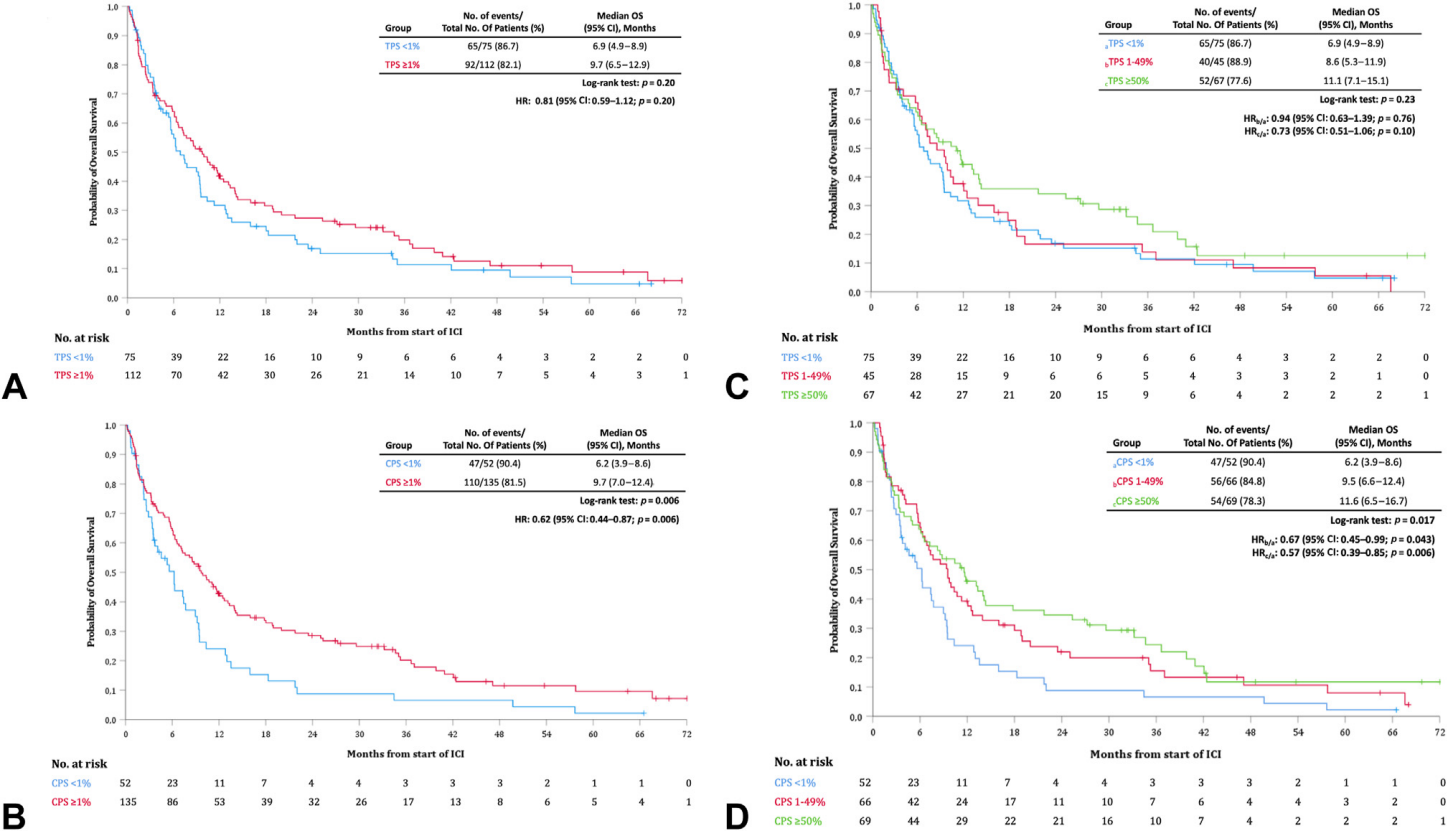
(*A*) Kaplan-Meier plot of OS for patients with TPS less than 1% (blue) and TPS more than or equal to 1% (red). (*B*) Kaplan-Meier plot of OS for patients with CPS less than 1% (blue) and CPS more than or equal to 1% (red). (*C*) Kaplan-Meier plot of OS for patients with TPS less than 1% (blue), TPS 1% to 49% (red), and TPS more than or equal to 50% (green). (*D*) Kaplan-Meier plot of OS for patients with CPS less than 1% (blue), CPS 1% to 49% (red), and CPS more than or equal to 50% (green). CPS, combined positive score; HR, hazard ratio; ICI, immune checkpoint inhibitor; OS, overall survival; TPS, tumor proportion score; CI, confidence interval.

The median OS from start of treatment was 6.9 months (95% CI: 4.9–8.9) in the TPS-negative group versus 9.7 months (95% CI: 6.5–12.9) in the TPS-positive group (HR = 0.81, 95% CI: 0.59–1.12, *p* = 0.20) (Fig. 4*A*). Figure 4*B* reveals a median OS of 6.2 months (95% CI: 3.9–8.6) in the CPS-negative group versus 9.7 months (95% CI: 7.0–12.4) in the CPS-positive group (HR = 0.62, 95% CI: 0.44–0.87, *p* = 0.006).

Figure 4*C* reveals a median OS of 6.9 months (95% CI: 4.9–8.9) versus 8.6 months (95% CI: 5.3–11.9) versus 11.1 months (95% CI: 7.1–15.1) in the TPS less than 1%, TPS 1% to 49%, and TPS more than or equal to 50% groups, respectively. When divided into CPS less than 1%, CPS 1% to 49%, CPS more than or equal to 50%, the median OS was 6.2 months (95% CI: 3.9–8.6) versus 9.5 months (95% CI: 6.6–12.4) versus 11.6 months (95% CI: 6.5–16.7), as found in Figure 4*D*.

**Figure 5:**
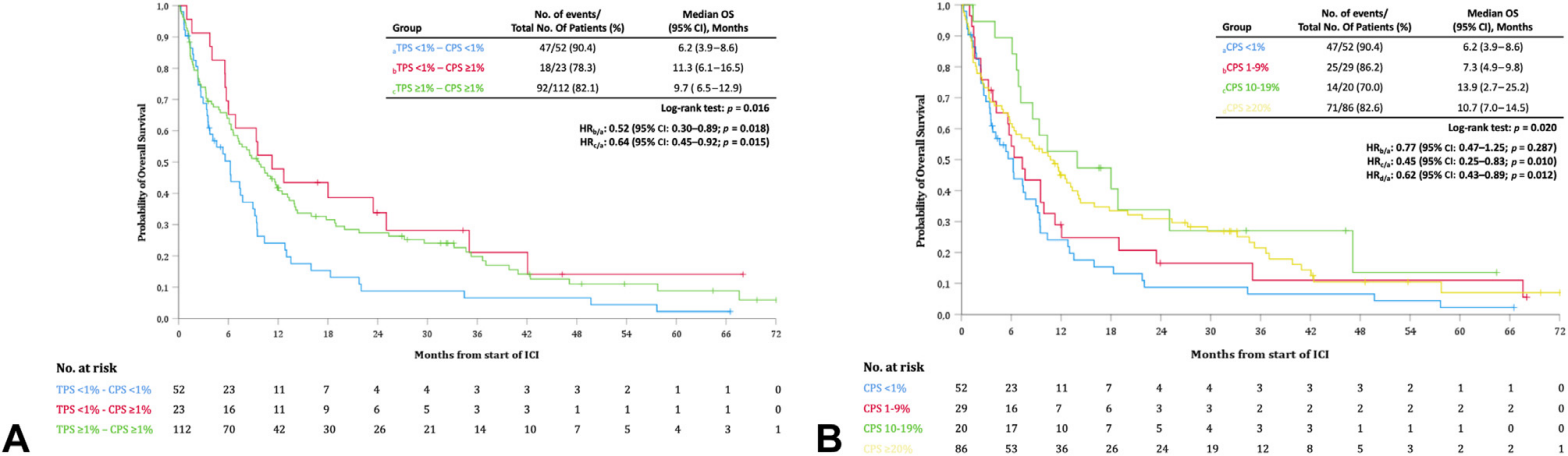
(*A*) Kaplan-Meier plot of OS for patients with TPS less than 1% and CPS less than 1% (blue), TPS less than 1% and CPS more than or equal to 1% (red), and TPS more than or equal to 1% and CPS more than or equal to 1% (green). (*B*) Kaplan-Meier plot of OS for patients with CPS less than 1% (blue), CPS 1% to 9% (red), CPS 10% to 19% (green), and CPS more than or equal to 20% (yellow). CPS, combined positive score; HR, hazard ratio; ICI, immune checkpoint inhibitor; OS, overall survival; TPS, tumor proportion score; CI, confidence interval.

The OS outcome in the TPS^−^/CPS^−^ subgroup (median of 6.2 mo; 95% CI: 3.9–8.6) was significantly shorter than those in the TPS^−^/CPS^+^ subgroup (median of 11.3 mo; 95% CI: 6.1–16.5, *p* = 0.018) and in the TPS^+^/CPS^+^ subgroup (median of 9.7 mo; 95% CI: 6.5–12.9, *p* = 0.015), as illustrated in Figure 5*A*. Using the four prespecified thresholds of CPS (<1%, 1%–9%, 10%–19%, ≥20%), the median OS was 6.2 months (95% CI: 3.9–8.6) versus 7.3 months (95% CI: 4.9–9.8) versus 13.9 months (95% CI: 2.7–25.2) versus 10.7 months (95% CI: 7.0–14.5), respectively (Fig. 5*B*). Separate OS analyses for first versus second line or beyond did not reveal significant differences (Supplementary Fig. 3*B* and *D*).

## Discussion

This single-center, retrospective study investigated the predictive value of PD-L1 positivity scored by CPS when compared with TPS in 187 patients with metastatic NSCLC treated with monotherapy immune checkpoint–based immunotherapy. To the best of our knowledge, this is the largest study reporting on the comparison of CPS and TPS in metastatic NSCLC.

Our results reveal that CPS could differentiate survival outcomes better than TPS. Indeed, using a PD-L1 positivity cutoff of 1%, only CPS could reveal a significant difference in OS and a positive trend in PFS. TPS, however, could not reveal a significant difference in both OS and PFS. Interestingly, the TPS-negative/CPS-positive (TPS^−^/CPS^+^) subgroup had a comparable OS with the TPS^+^/CPS^+^ subgroup.

The superiority of the CPS method in this study was mainly determined by the reallocation of the TPS^−^/CPS^+^ subgroup away from the TPS-negative subgroup, suggesting that this TPS^−^/CPS^+^ subgroup may have a comparable sensitivity to ICI as the TPS-positive subgroup through the PD-L1 blockade on tumor and inflammatory immune cells. The TPS^−^/CPS^+^ subgroup represented a substantial size as they accounted for approximately 30% among the patients with a negative TPS, which was approximately 12% in the total study population. Because of the scarcity of published data, we could not compare these frequencies with other external study data sets. Nevertheless, as we included all our eligible real-world institutional cases of metastatic NSCLC that were treated with ICI monotherapy in this study, we assume that these frequencies may approximate those in the general population and therefore represent a considerable amount of the overall patients with metastatic NSCLC treated with ICI.

PD-L1 scoring by CPS is being used in other cancer types such as head and neck squamous cell carcinoma and gastric carcinoma, on the basis of, among others, the results of the KEYNOTE-012 and KEYNOTE-055 studies, respectively.^7^^,^^8^ Conversely, in NSCLC, TPS is used as most ICI registration studies were performed with TPS as a diagnostic companion.^1^, ^2^, ^3^, ^4^ Remarkably, very limited data are available in the literature on the comparison of TPS and CPS in NSCLC. In a retrospective study performed by De Marchi et al.,^18^ investigating the use of CPS and TPS in 56 patients with NSCLC who received an ICI regimen including both monotherapy and combination therapies, the authors concluded that both TPS and CPS proved to be equally predictive of response to anti–programmed cell death protein 1/PD-L1 therapy. They saw a high intraobserver agreement between the two methods and a high interobserver agreement between pathologists and suggested that CPS could also be used in a routine setting for immunotherapy decision-making in NSCLC. In terms of clinical outcomes, the authors correlated CPS and TPS to radiologic response, OS, and post-ICI survival. Nevertheless, they did not report on the performance of the TPS^−^/CPS^+^ subgroup in comparison to other subgroups. In contrast, the present study, did find that CPS, mainly through the TPS^−^/CPS^+^ subgroup, could differentiate OS better than TPS.

This study has several strengths. First, its sample size is relatively large, allowing for a more granular assessment of the frequencies and the performance of different subgroups, namely the TPS^−^/CPS^+^ subgroup, which, to date, has not been reported on. In addition, a strict patient inclusion strategy was performed to include only those patients who received a monotherapy ICI treatment, avoiding the possible synergetic effects of combination therapies. Moreover, PD-L1 positivity was scored through both TPS and CPS methodologies by two experienced observers to prevent observer bias.

Nonetheless, several limitations also need to be considered. Although widely used as TPS in NSCLC, the predictive value of PD-L1 expression in terms of survival is not unquestioned.^19^ The evaluation of PD-L1 is a challenging assignment, especially when scored on immune cells, requiring appropriate training to ensure the results are reliable and reproducible. Besides, from an operational view, a number of confounding variables may be introduced by the biopsy sampling sites and the oldness of archival tissue specimens, including variations in other characteristics such as tumor histopathology and the different ICI agents that might have had an influence on survival outcomes.^20^, ^21^, ^22^ In this study, we did not report on the radiologic responses, as this was a retrospective study, and radiologic response metrics are best used with prospective designs that include fixed assessment time points. This study used the cutoff value of 1% for both TPS and CPS, based on literature. Nevertheless, it is not clear whether this is the most optimal cutoff value and should therefore be further explored in larger cohort studies. Finally, this was a single-center study, and these findings need to be validated in external cohorts.

In conclusion, this retrospective real-world study revealed that by using a cutoff value of 1%, CPS differentiated OS better than TPS in patients with metastatic NSCLC treated with monotherapy immune checkpoint-based immunotherapy. The difference between TPS and CPS was mainly determined by the TPS^−^/CPS^+^ subgroup, which was associated with a comparable survival outcome as the TPS^+^ subgroup. These results suggest that CPS may be a better predictive biomarker for ICI efficacy in patients with NSCLC, especially in those with a negative TPS. These findings need to be further substantiated in larger studies.

## CRediT Authorship Contribution Statement

**Ezgi B. Ulas:** Conceptualization, Data curation, Formal analysis, Investigation, Methodology, Project administration, Visualization, Writing—original draft.

**Sayed M.S. Hashemi:** Conceptualization, Data curation, Formal analysis, Investigation, Methodology, Project administration, Visualization, Writing—original draft.

**Ilias Houda:** Conceptualization, Data curation, Methodology, Project administration, Writing—original draft.

**Adem Kaynak:** Conceptualization, Data curation, Methodology, Writing—original draft.

**Joris D. Veltman:** Conceptualization, Methodology, Writing—review and editing.

**Marieke F. Fransen:** Conceptualization, Methodology, Writing—review and editing.

**Teodora Radonic:** Conceptualization, Methodology, Resources, Supervision, Writing—original draft.

**Idris Bahce:** Conceptualization, Methodology, Resources, Supervision, Writing—original draft.
