# Supplementary material for: Predictive Value of Combined Positive Score and Tumor Proportion Score for Immunotherapy Response in Advanced NSCLC
Source: JTO Clin Res Rep. 2023 May 25;4(9):100532. doi: 10.1016/j.jtocrr.2023.100532 (PMC10480627; doi:10.1016/j.jtocrr.2023.100532)
Supplement: Supplementary Tables and Figures [file mmc1.docx]

**Supplementary**

**Table 1.** Baseline clinicopathological characteristics of included patients with TPS-/CPS-, TPS-/CPS+ and TPS+/CPS+ baseline biopsies (n=52, n=23, n=112, respectively)

|  | TPS-/CPS- N (%) | TPS-/CPS+ N (%) | TPS+/CPS+ N (%) | P-value |  |
| --- | --- | --- | --- | --- | --- |
| Median age | 64 (37-78) | 63 (47-78) | 64 (32-89) | *0.79^X^* |  |
| Sex |  |  |  | *0.21^#^* |  |
| Male | 31 (59.6) | 13 (56.5) | 51 (45.5) |  |  |
| Female | 21 (40.4) | 10 (43.5) | 61 (54.5) |  |  |
| ECOG PS |  |  |  | *0.15^#^* |  |
| 0 | 9 (17.3) | 7 (30.4) | 22 (19.6) |  |  |
| 1 | 28 (53.9) | 12 (52.2) | 66 (58.9) |  |  |
| 2 | 14 (26.9) | 4 (17.4) | 13 (11.6) |  |  |
| 3-4-5 | 1 (1.9) | 0 (0.0) | 9 (8.0) |  |  |
| Unknown | 0 (0.0) | 0 (0.0) | 2 (1.9) |  |  |
| Smoking status |  |  |  | *0.82^#^* |  |
| Active | 14 (26.9) | 9 (39.1) | 39 (34.8) |  |  |
| Former | 33 (63.5) | 12 (52.2) | 62 (55.4) |  |  |
| Never | 2 (3.8) | 2 (8.7) | 8 (7.1) |  |  |
| Unknown | 3 (5.8) | 0 (0.0) | 3 (2.7) |  |  |
| Histologic type |  |  |  | *0.06^#^* |  |
| Adenocarcinoma | 35 (67.3) | 12 (52.2) | 74 (66.1) |  |  |
| Squamous cell carcinoma | 13 (25.0) | 7 (30.5) | 24 (21.4) |  |  |
| NOS | 0 (0.0) | 2 (8.7) | 10 (8.9) |  |  |
| LCNEC | 4 (7.7) | 1 (4.3) | 2 (1.8) |  |  |
| Sarcomatoïd carcinoma | 0 (0.0) | 0 (0.0) | 2 (1.8) |  |  |
| Adenosquamous carcinoma | 0 (0.0) | 1 (4.3) | 0 (0.0) |  |  |
| Immune checkpoint inhibitor |  |  |  | ***<0.001^#^*** |  |
| Nivolumab | 50 (96.2) | 21 (91.3) | 54 (48.2) |  |  |
| Pembrolizumab | 1 (1.9) | 1 (4.3) | 58 (51.8) |  |  |
| Atezolizumab | 1 (1.9) | 1 (4.3) | 0 (0.0) |  |  |
| Line of treatment |  |  |  | ***0.003^#^*** |  |
| First | 13 (25.0) | 6 (26.1) | 56 (50.0) |  |  |
| Second or beyond | 39 (75.0) | 17 (73.9) | 56 (50.0) |  |  |
| Site of origin |  |  |  | *0.50^#^* |  |
| Lung (primary) | 21 (40.4) | 13 (56.5) | 60 (53.6) |  |  |
| Lymph node | 8 (15.4) | 5 (21.7) | 10 (8.9) |  |  |
| Liver | 8 (15.4) | 1 (4.3) | 12 (10.7) |  |  |
| Soft tissue | 4 (7.7) | 0 (0.0) | 10 (8.9) |  |  |
| Bone | 5 (9.6) | 2 (8.7) | 3 (2.7) |  |  |
| Adrenal | 2 (3.8) | 0 (0.0) | 5 (4.5) |  |  |
| Pleura | 1 (1.9) | 0 (0.0) | 4 (3.6) |  |  |
| Cerebrum | 2 (3.8) | 1 (4.3) | 1 (0.9) |  |  |
| Lung (metastasis) | 0 (0.0) | 0 (0.0) | 2 (1.8) |  |  |
| Skin | 0 (0.0) | 0 (0.0) | 2 (1.8) |  |  |
| Other | 1 (1.9) | 1 (4.3) | 3 (2.7) |  |  |
| Abbreviations:  ECOG PS = Eastern Cooperative Oncology Group Performance Score; NOS = Not Otherwise Specified; LCNEC = Large Cell Neuro Endocrine Carcinoma; PD-L1 = Programmed Death-Ligand-1; N/A = Not applicable  ^x^ = Significance calculated by One-way ANOVA test  ^#^ = Significance calculated by Chi square (χ^2^) test | | | | | |

**Table 1 (continued).** Baseline clinicopathological characteristics of included patients with TPS-/CPS-, TPS-/CPS+ and TPS+/CPS+ baseline biopsies (n=52, n=23, n=112, respectively)

|  | TPS-/CPS- N (%) | TPS-/CPS+ N (%) | TPS+/CPS+ N (%) | P-value |  |
| --- | --- | --- | --- | --- | --- |
| Oncogenic drivers present |  |  |  | *N/A* |  |
| None | 15 (28.9) | 3 (13.0) | 20 (17.9) |  |  |
| TP53 | 16 (30.8) | 9 (39.1) | 42 (37.5) |  |  |
| KRAS | 13 (25.0) | 4 (17.4) | 40 (35.7) |  |  |
| STK11 | 1 (1.9) | 2 (8.7) | 3 (2.7) |  |  |
| FGFR1 amplification | 0 (0.0) | 2 (8.7) | 0 (0.0) |  |  |
| BRAF | 3 (5.8) | 2 (8.7) | 9 (8.0) |  |  |
| EGFR | 1 (1.9) | 1 (4.3) | 1 (0.9) |  |  |
| MET mutation | 0 (0.0) | 1 (4.3) | 5 (4.5) |  |  |
| Unknown | 7 (13.5) | 7 (30.4) | 12 (10.7) |  |  |
| Abbreviations:  ECOG PS = Eastern Cooperative Oncology Group Performance Score; NOS = Not Otherwise Specified; LCNEC = Large Cell Neuro Endocrine Carcinoma; PD-L1 = Programmed Death-Ligand-1; N/A = Not applicable  ^x^ = Significance calculated by One-way ANOVA test  ^#^ = Significance calculated by Chi square (χ^2^) test | | | | | |

**Table 2.** Interobserver agreement analysis by Kappa analysis in a test cohort (N = 50)

|  |  | Observer A | Observer B | Cohen’s Kappa coefficient | 95% CI | P-value |
| --- | --- | --- | --- | --- | --- | --- |
| TPS | <1% | 21 (42%) | 23 (46%) | 0.789 | 0.660 – 0.918 | <0.001 |
|  | 1-49% | 3 (6%) | 1 (2%) |  |  |  |
|  | ≥50% | 26 (52%) | 26 (52%) |  |  |  |
| CPS | <1% | 14 (28%) | 14 (28%) | 0.707 | 0.572 – 0.842 | <0.001 |
|  | 1-49% | 8 (16%) | 9 (18%) |  |  |  |
|  | ≥50% | 28 (56%) | 27 (54%) |  |  |  |

**Figure 1.**

A. Kaplan-Meier plot of PFS for patients with TPS <1% (blue) and TPS ≥1% (red).

B. Kaplan-Meier plot of PFS for patients with CPS <1% (blue) and CPS ≥1% (red).

C. Kaplan-Meier plot of PFS for patients with TPS <1% (blue), TPS 1-49% (red) and TPS ≥50% (green).

D. Kaplan-Meier plot of PFS for patients with CPS <1% (blue), CPS 1-49% (red) and CPS ≥50% (green).


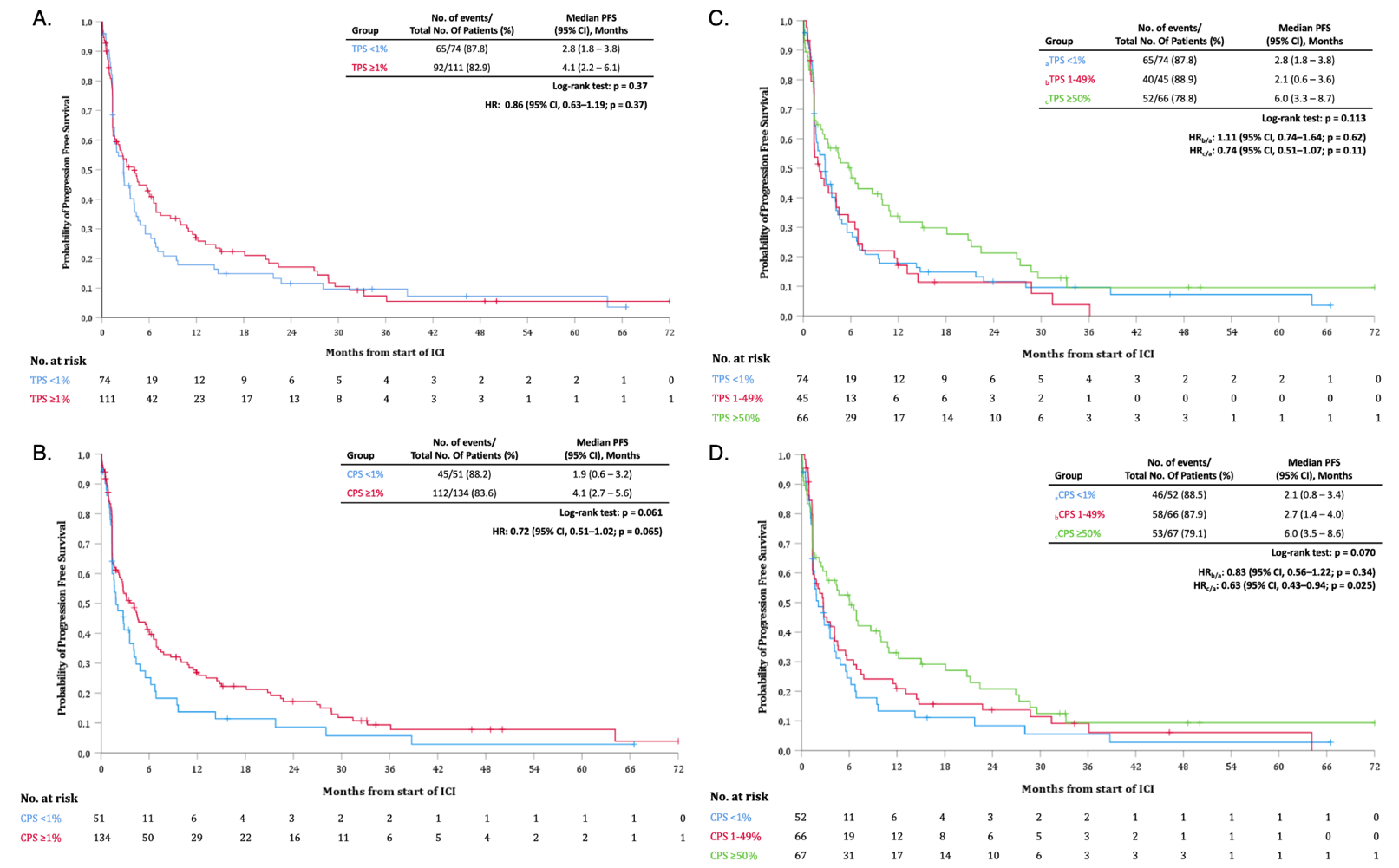


**Figure 2.**

A. Kaplan-Meier plot of PFS for patients with TPS <1% and CPS <1% (blue), TPS <1% and CPS ≥ 1% (red) and TPS ≥1% and CPS ≥1% (green).

**
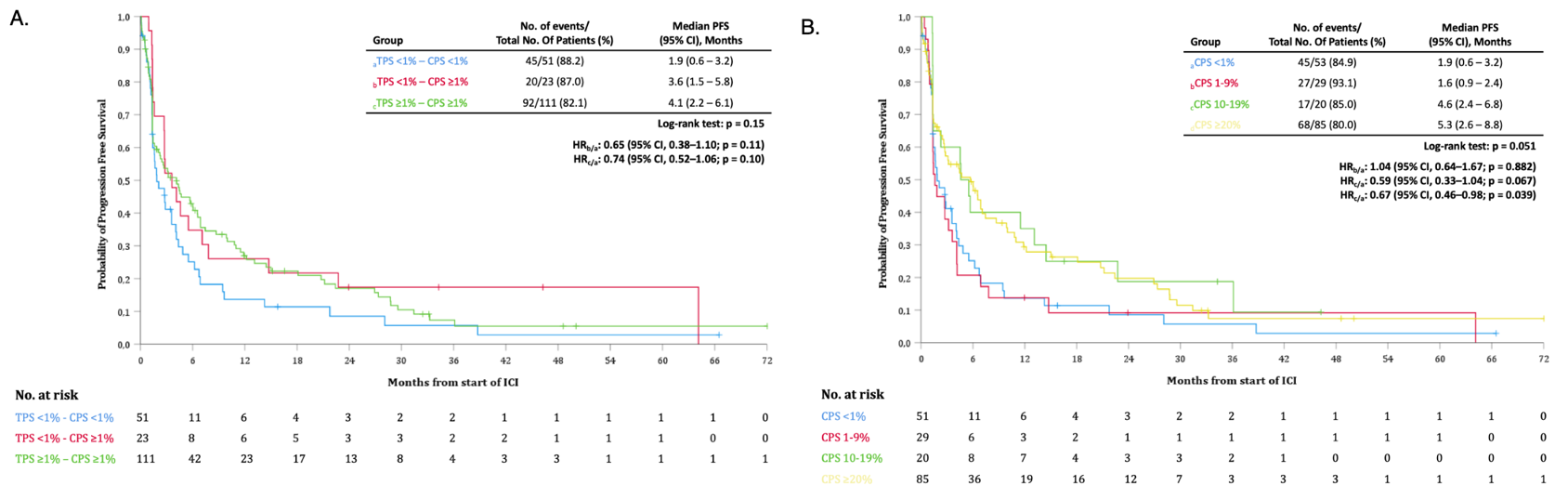
**B. Kaplan-Meier plot of PFS for patients with CPS <1% (blue), CPS 1-9% (red), CPS 10-19% (green) and CPS ≥20% (yellow).

**Figure 3.**

A. Kaplan-Meier plot of PFS for patients with CPS <1% (blue) and CPS ≥1% (red) that received first-line ICI treatment.

B. Kaplan-Meier plot of OS for patients with CPS <1% (blue) and CPS ≥1% (red) that received first-line ICI treatment.

C. Kaplan-Meier plot of PFS for patients with CPS <1% (blue) and CPS ≥1% (red) that received ICI treatment in second line or beyond.


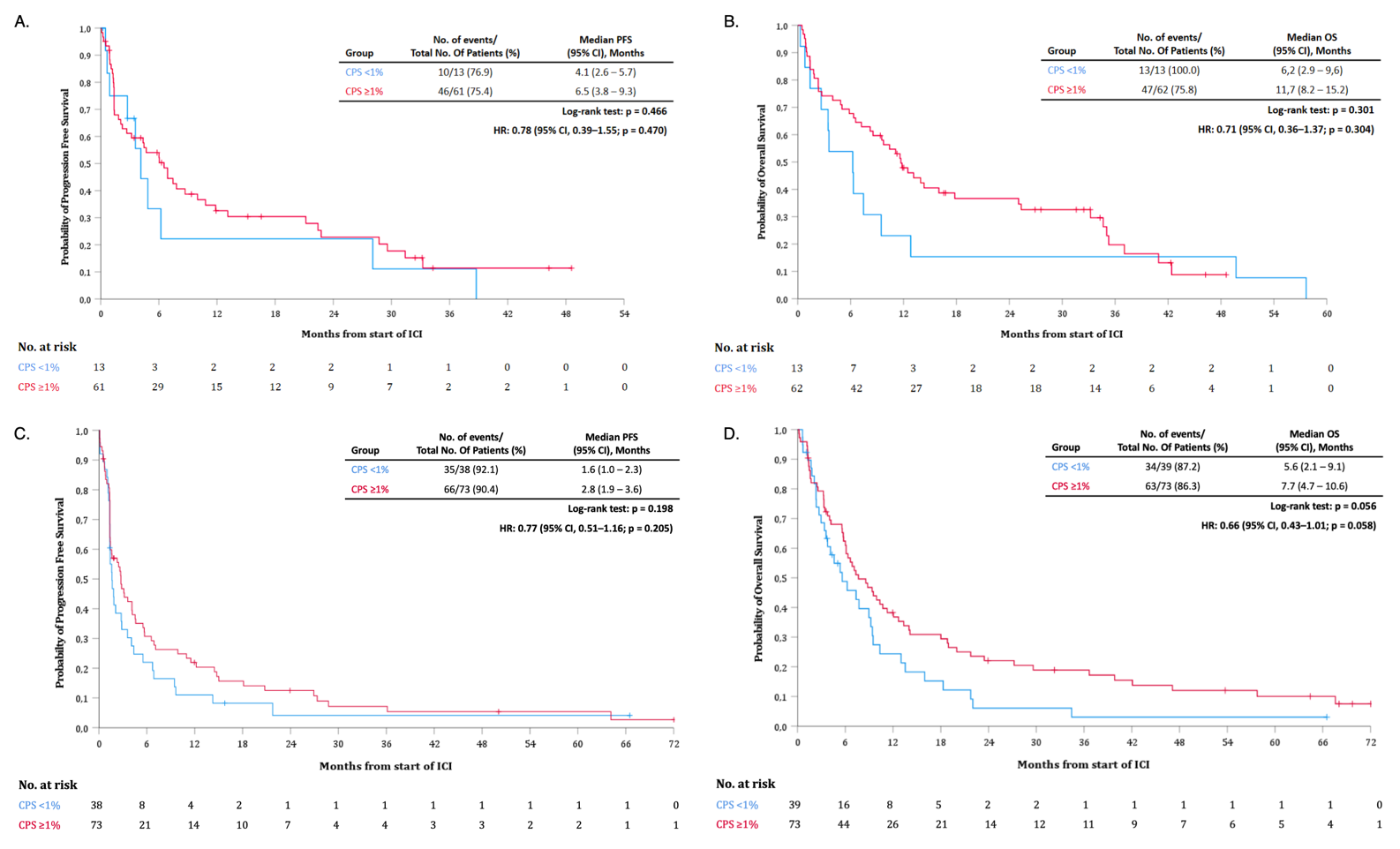
D. Kaplan-Meier plot of OS for patients with CPS <1% (blue) and CPS ≥1% (red) that received ICI treatment in second line or beyond.

**Supplementary Methods**

1. **PD-L1 staining by Immunohistochemistry**

Deparaffination/antigen retrieval was done with Low pH buffer for 20 minutes at 97°C in a PT module. Other steps were done in the Dako Autolink 48 IHC staining device at room temperature. Incubation with PD-L1 clone 22C3 1/55 in Dako diluent S3022 was performed for 30 minutes. Detection with FLEX mouse linker as well as with FLEX Envision-HRP was done for 30 minutes. Visualization with FLEX DAB plus for 10 minutes and DAB Enhancer for 5 minutes. Afterwards the slides were counterstained with hematoxylin and rinsed with running tapwater. Slides were dehydrated with Ethanol 100%, cleared with Xylene and mounted with TissueTEK II film (Sakura).
